# Effect of Kinetic Degrees of Freedom on Multi-Finger Synergies and Task Performance during Force Production and Release Tasks

**DOI:** 10.1038/s41598-018-31136-8

**Published:** 2018-08-24

**Authors:** Kitae Kim, Dayuan Xu, Jaebum Park

**Affiliations:** 1 Department of Physical Education, Seoul National University, Seoul, South Korea; 2 Institute of Sport Science, Seoul National University, Seoul, South Korea

## Abstract

Complex structures present in a human body has relatively large degrees-of-freedom (DOFs) as compared to the requirement of a particular task. This phenomenon called motor redundancy initially deemed as a computational problem rather can be understood as having the flexibility to perform the certain task successfully. Hence, the purpose of our study was to examine the positive impact of extra DOFs (redundant DOFs) during force production tasks. For this purpose, an experimental setup was designed to simulate archery-like shooting, and purposeful organization of a redundant set of finger forces determined the stability of important performance variables as well as accurate and precise performance. DOFs were adjusted by changing the number of fingers explicitly involved in the task. The concept of motor synergy and computational framework of uncontrolled manifold (UCM) approach was used to quantify stability indices during finger force production. As a result, accuracy and precision of the task improved with an increase in DOFs. Also, the stability indices of net finger forces and moment increased with active DOFs of fingers. We concluded that the controller actively utilizes extra DOFs to increase the stability of the performance, which is associated with the improved accuracy and precision of the task.

## Introduction

The human body is a complex system of redundant forms in which the degrees-of-freedom (DOFs) of elements is generally larger than the DOFs (i.e., dimensionality) of task space, and this phenomenon has been postulated as the motor redundancy or the DOF problem^1,2^. The investigation on the strategies to govern a redundant set of elements in humans has been one of the most challenging issues, and no consensus has been made on the issue of the controller’s strategy to solve the redundancy. From the engineering or mathematical point of view, the redundancy has been considered a negative aspect of the system due to a computational difficulty on the control system. Therefore, several studies have been conducted to formulate computational models that examined an optimum solution by means of eliminating, freezing^3^, or coupling a redundant set of DOFs^4–6^. In the optimization of the redundant system, the existence of variability (or inconsistent combinations of elements) could reflect an erroneous neural process. An alternative idea suggests that the human controller utilizes redundant DOFs positively in such a way that the elements show purposeful covariations to satisfy a particular motor task resulting in forming a family of solutions as to portray flexible solutions. Also, the idea assumes that the variability within the family of solutions may be an indicator of the control process since there are task-related rules to form the family of solutions^7^. Thus, specific performance could be executed successfully in a flexible manner while having a purposeful organization of elements. Indeed, experimental evidence has supported that the various combinations of elements equally satisfy a specific motor performance and the organization of flexible combinations of the elements depends on either mechanical necessities of the motor tasks^8,9^ or choice of the controller for maintaining the stability of performance variables^9–11^. Stability in traditional terms refers to the ability of the system to return to its original state in response to external perturbations^12^. In the same context, the stability in the redundant system of human movements describes the ability to stabilize a specific performance variable by flexible sharing and error compensation among elements in task-specific manners^13,14^.

The system of human hands and fingers has been modeled as a serial and parallel system, and the redundancy of the human hand system is observed through both kinematic and kinetic variables. The previous studies regarding the solutions to the kinetic redundancy of the human hand system reported that individual finger forces co-vary to stabilize important performance variables that produces desired net mechanical outcomes such as net force or net moment of force^9,11,15–18^. On the other hand, a recent study of finger force organization during pressing task quantified and compared optimal combinations and variability associated with finger forces, which showed that the optimal patterns of finger forces and their variance components are compatible^19^. During a multi-finger pressing task, covariation patterns for the net torque stabilization is in conflict with the covariation pattern for the force stabilization if only normal forces of two fingers were considered while the two finger forces produced opposite directional torques to each other^9^. However, it is theoretically possible to stabilize both net force and moment simultaneously with the usage of more than two fingers, and a possible range of solutions relies on the number of redundant fingers involved in the task. Therefore, it may be advantageous to have a redundant set of fingers in producing and maintaining a certain amount of the net force and torque simultaneously with a more flexible pattern of finger force combinations. This expectation and experimental observation are in line with the concept of motor abundance^2,20^. Hence, “*Redundant*” elements may not cause computational problem or errors of the neuronal process. Rather, the system with redundant elements could be viewed as a prerequisite for flexibility (or stability) upon a proper organization of finger actions. The strategies of organizing family of solutions for successful performance is definitely a process of neural structure in the biological system, and term, synergy, has been proposed to describe and quantify the process and consequence of neural activities for governing a redundant set of finger forces. For the quantification of synergic actions of elements, the framework of the uncontrolled manifold (UCM) approach has been proposed^21,22^. The process includes selecting two lower dimensional subspaces within the space of elements. The first subspace is concerned with the manifold where the changes in the actions of elements have no net mechanical effect, and the second subspace is the orthogonal space to the UCM where the actions of elements do have net mechanical effect. If most of the force variance of individual fingers is confined within the UCM (i.e., V_UCM_) and the variance observed in the orthogonal space to the UCM (i.e., V_ORT_) is relatively small, we may conclude that the net force is stabilized by the covaried adjustment of individual finger forces.

Here, we would like to raise two follow-up questions regarding the organization of a redundant set of elements during multi-finger force production tasks. The first question is “*How many kinetic DOFs of fingers would be best to stabilize net force and torque during multi-finger pressing task?*” The second question is “*Is the synergy beneficial to improve accuracy and precision of performance?*” A shooting task could exemplify the topics raised by two questions, and the archery shooting with multiple combinations of fingers is a good candidate for motor tasks to examine the effect of kinetic DOFs and its further effect on the shooting performance. Apparently, successful shooting performance in the archery shooting depends on accurate and stable force production by multi-fingers during aiming, and quick release of the net finger force during force release to minimize the changes in momentum^23^, which results in “*good*” shooting performance consistently. The terms, accuracy and precision, have been used to quantify the indices of the shooting performance^23–25^. The accuracy refers to the closeness of performed values to a required value, and the precision refers to the reproducibility of performances.

In the current study, we employed a multi-finger pressing task with a set of finger combinations (i.e., a set of DOFs of fingers) simulating the shooting motion of the archery. The main goal of the current study was to explore the effect of kinetic DOFs (i.e., number of involved fingers as force generators) on the stabilization of the total force (F_TOT_) and moment of force (M_TOT_, i.e., the product of finger forces and their lever arms) during stable force production. Further, we examined how the stability properties of multi-finger actions during a steady-state force production affect the shooting performance such as accuracy and precision. We tested the following hypotheses: (1) the synergy indices of both total force and moment will increase with the number of fingers during a steady-state force production, and (2) the accuracy and precision as the indices of shooting performance will increase (i.e., improved accuracy and precision) with the number of fingers.

## Results

The motor task in the current study imitated the finger actions of the archery in such a way that the participant produced a steady-state value of finger force followed by the release of the force quickly. In addition, the participants performed the tasks with a set of finger combinations that was incorporated with the kinetic DOFs of actively involved fingers during the tasks. Shooting performance was quantified by the indices of accuracy and precision that refer to the distance to the target value and the reproducibility of repetitive attempts, respectively. The framework of uncontrolled manifold analysis was used to quantify force variance within two subspaces of finger forces and the synergy indices as a stability property of net force and moment production. Further, we examined correlations between the indices of task performance and multi-finger synergy indices. See the Methods section for more details.

### Performance indices

#### Root mean squared error (RMSE)

**Figure 1:**
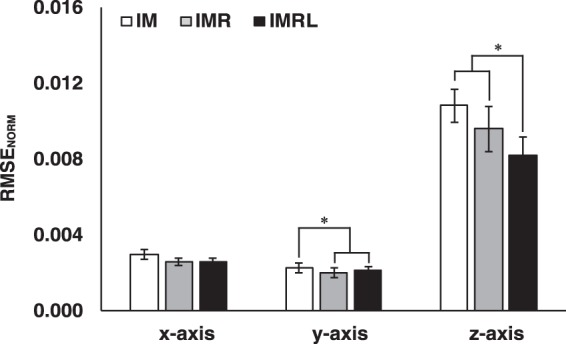
RMSE_NORM_ at the steady-state force production for the *x*-, *y*-, and *z*-axis are presented for the IM (white bars), IMR (gray bars), and IMRL conditions (black bars). Values are means ± standard errors across subjects. The *asterisks* show significant differences in pairwise comparisons between the conditions (*p* < 0.05).

The root mean squared error normalized by reference force (RMSE_NORM_) during a steady-state force production was relatively larger in the *z*-axis (i.e., the axis of normal force) compared to the RMSE_NORM_ of the other two axes. In addition, the RMSE_NORM_ decreased with the number of fingers (DOFs) in the *y*-axis (superior-inferior) and *z*-axis. The RMSE_NORM_ of the *x*-axis (medial-lateral) was similar between the conditions (Fig. 1). A two-way repeated measure ANOVA supported these findings with factors *Axis* (three levels: *x*-, *y*-, and *z*-axis) and *Finger DOF* (three levels: IM, IMR, and IMRL), which showed significant main effects of *Axis* (*F*_[0.08,7.57]_ = 77.97, *p* < 0.001, *ηp²* = 0.92) and *Finger DOF* (*F*_[2,14]_ = 5.65, *p* = 0.02, *ηp²* = 0.54) with a significant *Axis* × *Finger DOF* (*F*_[1.80,12.58]_ = 8.70, *p* < 0.001, *ηp²* = 0.55). A significant factor interaction reflected the fact that a significant effect of *Finger DOF* was observed only in the *y*- and *z*-axis. Pair-wise comparison confirmed that the RMSE_NORM_ of IM > IMR, IMRL for the *y*-axis (*p* < 0.05) and IM, IMR > IMRL for the *z*-axis (*p* < 0.05).

#### Release time (RT)

The participants performed 25 repetitive trials for the multi-finger force production and release (FPR) task for each DOF condition. Generally, there was no significant difference in the average release time across the trials (RT_AVG_) between the conditions. However, the standard deviation of RT (RT_SD_) decreased with the number of fingers. In other words, the RT was more consistent when the number of involved fingers was increased. A one-way repeated measure ANOVA with factor *Finger DOF* (three levels: IM, IMR, and IMRL) on RT_SD_ supported these findings (*F*_[2,14]_ = 5.42, *p* = 0.02, *ηp²* = 0.44). Post-hoc pairwise comparisons confirmed RT_SD_ of IM, IMR > RT_SD_ of IMRL (*p* < 0.05).

#### Accuracy index (ACI) and precision index (PRI)

The indices of accuracy (ACI) and precision (PRI) decreased together with the number of finger DOFs (i.e., the larger the number of fingers for the task, the better accuracy and precision for the performance). These findings were supported by one-way repeated measure ANOVAs with factor *Finger DOF* (three levels: IM, IMR, and IMRL) separately on ACI (*F*_[1.10,7.72]_ = 57.53, *p* < 0.001, *ηp²* = 0.89) and PRI (*F*_[2,14]_ = 29.73, *p* < 0.001, *ηp²* = 0.81). Post-hoc pairwise comparisons confirmed that the IM > IMR and IMRL for ACI (*p* < 0.05), and the IM > IMR > IMRL for PRI (*p* < 0.05).

### Multi-finger synergy indices

#### Force stabilization hypothesis

**Figure 2:**
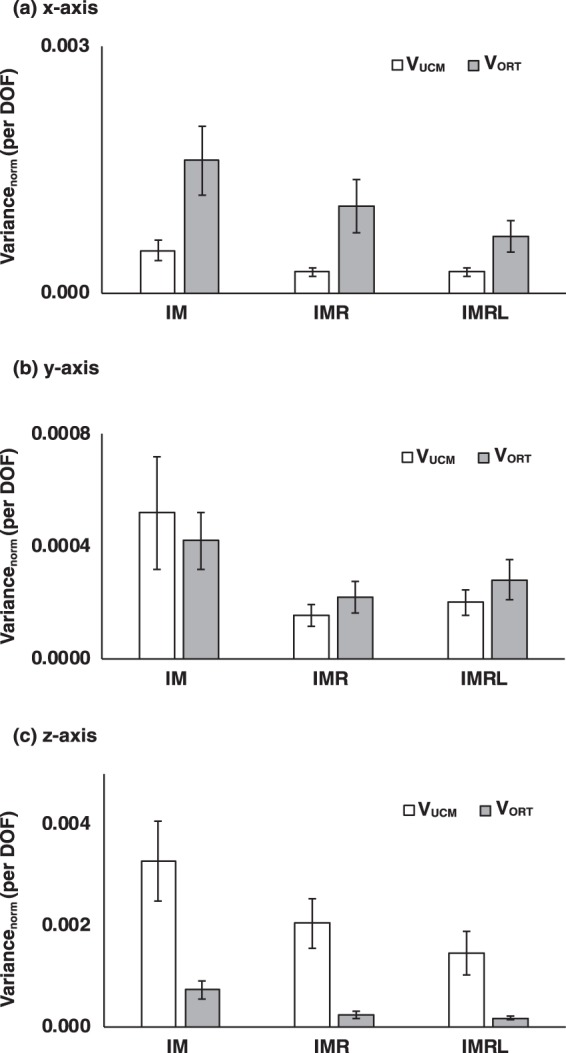
Two components of variances related to F_TOT_ stabilization, V_UCM_ (white bars) and V_ORT_ (gray bars) per degree-of-freedom, in the finger force space are presented for the (**a**) *x*-axis, (**b**) *y*-axis, and (**c**) *z*-axis. Values are means ± standard errors across subjects.

First, we compared mean differences of each component of variances per DOF for the force stabilization hypothesis (V_UCM_^F^ and V_ORT_^F^) between the conditions and axes. The variances were normalized by the square of the relevant reference force (F_REF_) about the *z*-axis. In general, both V_UCM_^F^ and V_ORT_^F^ decreased with the number of finger DOFs for all three axes. In particular, V_UCM_^F^ was smaller than V_ORT_^F^ in the *x*-axis, while V_UCM_^F^ was larger than V_ORT_^F^ in the *z*-axis (axis of normal force) (Fig. 2). Two-way repeated measure ANOVAs with factors *Finger DOF* (three levels: IM, IMR, and IMRL) and *Variance* (two levels: UCM and ORT) were performed separately on the variances of each axis. The results showed significant main effects of *Finger DOF* (*x*-axis: *F*_[2,14]_ = 7.66, *p* = 0.01, *ηp²* = 0.52; *y*-axis: *F*_[1.16,8.11]_ = 5.13, *p* = 0.05, *ηp²* = 0.42; *z*-axis: *F*_[2,14]_ = 6.53, *p* = 0.01, *ηp²* = 0.48) and *Variance* (*x*-axis: *F*_[1,7]_ = 9.52, *p* = 0.02, *ηp²* = 0.58; *z*-axis: *F*_[1,7]_ = 6.53, *p* < 0.01, *ηp²* = 0.78) without factor interactions.

**Figure 3:**
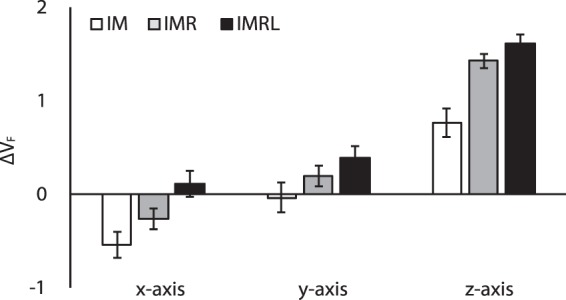
Z-transformed synergy indices with respect to F_TOT_ stabilization, ΔV_F_, for the IM (white bars), IMR (gray bars), and IMRL conditions (black bars) at the *x*-, *y*-, and *z*-axis. Values are means ± standard errors across the subjects.

Further, we quantified the indices of synergies for the force stabilization (ΔV_F_) during the steady-state force production. Overall, ΔV_F_ increased with the number of finger DOFs for all three axes, and the stabilization of normal force (ΔV_F_^Z^) was stronger than the stabilization of tangential forces (ΔV_F_^X^ and ΔV_F_^Y^) (Fig. 3). These findings were supported by a two-way repeated measure ANOVA on ΔV_F_ setting factors as *Finger DOF* (three levels: IM, IMR, and IMRL) and *Axis* (three levels: *x*-, *y*-, and *z*-axis). The results showed significant main effects of *Finger DOF* (*F*_[1.21,8.49]_ = 40.23, *p* < 0.001, *ηp²* = 0.85) and *Axis* (*F*_[2,14]_ = 74.89, *p* < 0.001, *ηp²* = 0.92) without factor interactions. Post-hoc pairwise comparison confirmed ΔV_F_ of IM < IMR < IMRL (*p* < 0.05), and ΔV_F_ of the *x*-axis < *y*-axis < *z*-axis (*p* < 0.05).

#### Moment stabilization hypothesis

**Figure 4:**
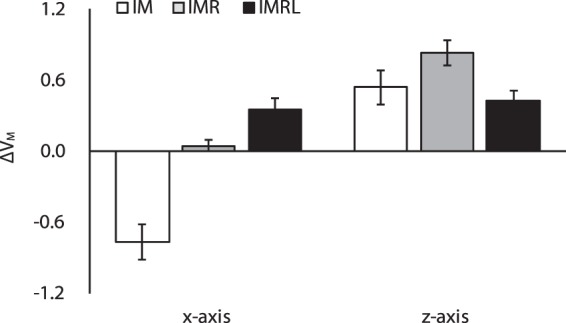
Z-transformed synergy indices related to M_TOT_ stabilization, ΔV_M_, for the IM (white bars), IMR (gray bars), and IMRL conditions (black bars) at the *x*- and *z*-axis. Values are means ± standard errors across the subjects.

We computed the synergy indices of the moment of force about the *x*- (ΔV_M_^X^) & *z*-axis (ΔV_M_^Z^) with the assumption of fixed moment arms for individual fingers. Note that the moment of force about the *y*-axis was zero due to the zero moment arm about the *x*- & *y*-axis. During the steady-state force production, ΔV_M_ increased with the number of fingers as the results of ΔV_F_ showed. In particular, the average ΔV_M_^X^ across the participants was negative in the IM condition, while it became positive in the IMRL condition (Fig. 4). ΔV_M_^Z^ was positive for all DOF conditions. A two-way repeated measure ANOVA on ΔV_M_ with factor *Finger DOF* (3 levels: IM, IMR, and IMRL) and *Axis* (three levels: *x*-, and *z*-axis) confirmed a significant main effect of *Finger DOF* (*F*_[2,14]_ = 14.25, *p* < 0.001, *ηp²* = 0.67) and *Axis* (*F*_[1,7]_ = 91.28, *p* < 0.001, *ηp²* = 0.93) with factor interactions (*F*_[2,14]_ = 24.98, *p* < 0.001, *ηp²* = 0.78). Post-hoc pairwise comparison confirmed ΔV_M_^X^ of IM < IMR < IMRL (*p* < 0.05) and ΔV_M_^Z^ of IM, IMRL < IMR (*p* < 0.05).

### The comparison between synergy indices and performances indices

**Figure 5:**
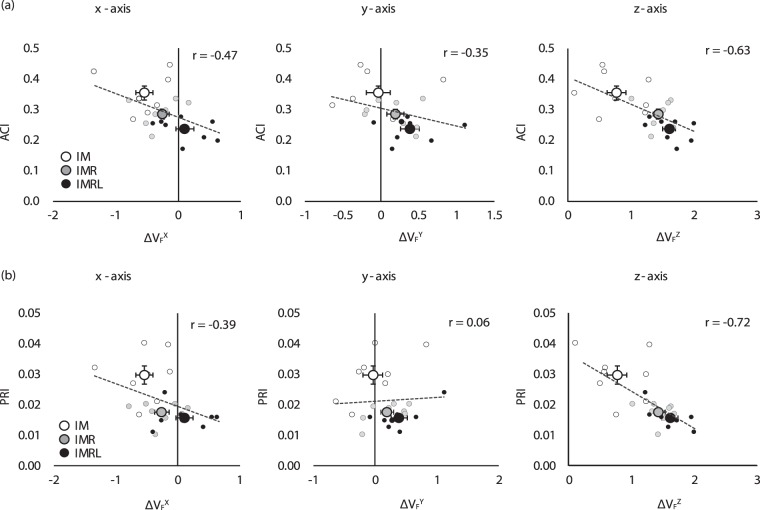
Correlation between the synergy indices of F_TOT_ stabilization for three axes (i.e., ΔV_F_^X^, ΔV_F_^Y^, and ΔV_F_^Z^) and (**a**) accuracy index (ACI) and (**b**) precision index (PRI). Small dots represent individual subject data for the IM (white), IMR (gray), and IMRL (black) conditions. The regression lines are shown with the coefficient of determination (*r*-value). The average values across subjects for the IM (white), IMR (gray), and IMRL (black) condition are presented with standard error bars in large circles.

**Figure 6:**
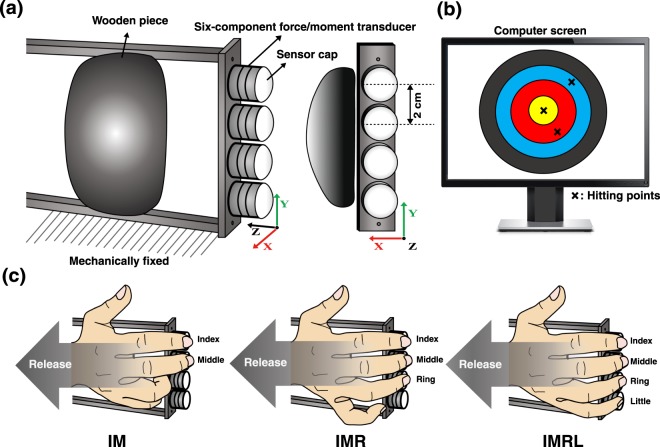
An illustration of the experimental equipment and conditions. (**a**) The experimental frame was mechanically fixed to the table and the sensors were attached to the experimental frame (size: 90 × 140 × 250 mm). The uncertainty values of the transducers as the validity of measurement were measured. The estimated results show the measurement errors ranged from 0.02–0.05 N for the force and 0.02–0.06 Ncm for the moment of force, which are compatible with the values reported in the previous studies^43,44^. Finely round-shaped caps were inserted into the surface of the transducers, which allowed fingertips to have intentional roll or slide naturally. The distance between adjacent transducers was set at 2 cm along the y-axis, and a wooden piece was placed under the palm to ensure a consistent configuration of the hand and fingers during the tasks^45^. The sampling rate of the force signals was set at 100 Hz. A 27-inch computer screen was positioned in front of the subject, which provided real-time force feedback. The refresh rate of the feedback screen was 60 Hz. (**b**) The computer screen displayed a hitting point of the virtual ball after the completion of a particular trial. The computer screen showed F_TOT_ produced by the participants and F_REF_. After the completion of a particular trial, the computer screen showed a hitting point of the virtual ball by assuming that the trajectory of the ball was determined by the velocity at the moment of release and gravitational force (*g* = 9.81 m/s^2^) acting on the ball. (**c**) Hand and finger configurations for three experimental conditions. The thumb was parallel to the fingers and naturally flexed the proximal interphalangeal joint to 10–20°. Similarly, non-involved fingers in the IM and IMR conditions were also flexed naturally while not contacting transducers; therefore, the thumb and non-involved finger(s) had no mechanical effect on the frame during the task.

Figure 5 illustrates the findings for the sets of synergy indices (e.g., ΔV_F_^X^, ΔV_F_^Y^, and ΔV_F_^Z^) and the variables associated with the performance indices (e.g., ACI and PRI) across all individual subjects and conditions. For most cases, there were significant negative correlations between the synergy indices and performance variables (e.g., ACI and PRI) across subjects and conditions (i.e., the larger synergy indices, the better accuracy and precision). In particular, the coefficient of correlation (*r*) was larger in ΔV_F_^Z^ vs. both ACI and PRI than in the synergy indices of the other axes vs. the performance indices (ΔV_F_^Z^: *r* = −0.63 for ACI, *r* = −0.72 for PRI; ΔV_F_^X^: *r* = −0.47 for ACI, *r* = −0.39 for PRI; ΔV_F_^Y^: *r* = −0.35 for ACI, *r* = 0.06 for PRI, *p* < 0.05 for all comparisons except ΔV_F_^Y^ vs. PRI). Further, the three large points in Fig. 6 present the overall mean values of the synergy and performance indices across the subjects within each experimental condition (e.g., IM, IMR, or IMRL). The mean values were aligned along negative straight lines in most cases. These results reflect that the increase in synergy indices with larger DOFs of fingers was closely related to the increase in accuracy and precision of the performance (i.e., decrease in ACI and PRI).

## Discussion

We tested two hypotheses formulated in the Introduction, and all leading hypotheses have been confirmed. The strength of synergy indices for both net force and moment increased with the number of fingers during the steady-state force production. Also, the accuracy and precision as the indices of shooting performance improved with the number of fingers. Further, the results showed that the indices of shooting performance were significantly correlated with the strength of stability properties (i.e., synergy indices) of both force and moment stabilization and their changes with the kinetic DOFs.

According to the principle of motor abundance^2,20^, flexible combinations of finger forces within a redundant set of elements to the task mechanics could be interpreted as the strategies employed by the controller since the organization of force variability is not uniform for every task. Preferably, the patterns of variability (i.e., covariation) of elements comply with a set of rules considering at least mechanical necessities of the task. Indeed, the relatively large across-trial variance is generally observed in the subspace where equal performance (i.e., mechanical necessity) is achieved compared to the variance observed in the orthogonal space to it. Thus, the underlying mechanism of the organization of variability may include purposeful flexibility to satisfy the task performance and to minimize the performance error, which is well fit with the idea of stochastic optimal control model^26^. The results of current study showed that the redundant set of fingers affects positive changes in the net force error to the target value by showing that the net force error (e.g., RMSE_NORM_) and error variance (V_ORT_) were the smallest when all four fingers were involved. The small error observed in the four fingers should positively affect the accuracy and precision of the performance as the experiment was designed. Particularly, the precision of the performance across multiple trials assumes to be related to the standard deviation of the release time. In other words, the increase of the finger DOFs seems to be beneficial in reproducible release time across trials, which yields higher precision of the performance.

The rules mentioned above describe the flexible sharing and covariation between redundant elements for error compensation resulting in the stabilization of salient performance variable (e.g., net finger force). Here, we would like to emphasize that the flexibility is not a prerequisite for the smaller performance error. This means that the consistent combinations of finger forces could also be the solution for small performance error, which is assumed to be a unique optimal solution. Thus, we can say that the net force error in the current results could be combined with flexible combinations of finger forces for the purpose of error compensation among active elements. Now, we come back to the questions of “*How many kinetic DOFs of fingers would be the best to stabilize net force and torque during multi-finger force production task?*” We know the fact that the middle and little fingers are relatively weak and less independent in their force production^27^ as well as the motion of joints^28^. Also, the damping ratio of the two fingers, which assumes to be indicative of stability, is smaller than that of the other two fingers^29^. So, one may think that the use of those two fingers is not helpful to improve performance and stability. However, the experimental evidence is opposite to the expectation. The previous and current results showed that the involvement of those two fingers was not detrimental, but somewhat beneficial to the overall performance and stabilization of the performance. These outcomes could also be associated with the results of a recent study about the effect of fatigue, which showed that the interaction of individual effectors reduces the error caused by the fatigued element^13,14^. This can be evidence that the controller is actively using redundant DOFs of multi-elements by minimizing performance error.

The explicit external constraint in the current experiment was to produce equal net finger forces to target forces, and the net moments was not mechanically constrained. The results showed that both net pressing force and corresponding moment of pressing force were stabilized especially when all four fingers were explicitly involved. If a set of the mechanical constraints in motor task was to satisfy both net force and moment constraints simultaneously, and only normal components of finger forces were considered with fixed fingertip contacts, two subspaces spanned by the eigenvectors of the Jacobians of the two motor tasks are orthogonal to each other. The intersection between the two subspaces confines the solutions of finger forces for satisfying the mechanics of net force and moment. With the case of two normal finger forces with two task constraints, a unique combination for finger forces satisfying the two tasks (i.e., optimal combination) could be set since the intersection between the two subspaces is not space, but a point, so that the redundancy is disappeared. If the system is redundant, however, the intersection forms space, and the dimensionality of the intersection space (i.e., solution space) depends on the number of redundant DOFs. This implies that the variability of finger force combinations is feasible observation, which satisfies the two constraints. By combining this knowledge with the current results, it is possible that the additional constraints (or satisfaction) about the moment stabilization may be embedded in the hand action and its control when the system has relatively large redundancy. Indeed, it is well discovered that the apparent task mechanics (e.g., net force production task) does not exclusively define actual variables to be stabilized selectively^8,30^. Maybe, the variables actually being controlled are not cognitively recognized, but are the part of the controller’s consideration to stabilize something else. It has been reported that both resultant force and moment of force could be stabilized with sufficient active elements in multi-digit pressing and grasping tasks^31–33^. Further, the resultant force and the moment of force by fingers were stabilized together even when moments are not provided as feedback^8,30^.

The elemental variables in the current analysis were the finger forces, and it is well known that the production of four finger forces is interdependent due to the phenomenon of enslaving (i.e., unintended force production or motion by non-task digit). The finger force enslaving is caused by two groups of factors that include the mechanical coupling of finger actions by passive connection and the overlapped representation of the digits in the hand area within the primary motor cortex^27^. Due to these factors of the enslaving, the structure of observed variances of mechanical variables (e.g., finger forces) may not definitely reflect the particular control strategy for a specific task. Instead of using interdependent mechanical variables, the variables that reflect the scheme of the controller have been introduced, and the variables are termed as force modes (Latash *et al*.^15^ for more details about force mode). A set of the elemental variables employed in the current analysis was finger forces in both normal and shear directions. It has been shown that the enslaving patterns of tangent directional forces are not as simple as those of normal forces, and particular relations between the interdependency of normal and tangential forces has not been discovered yet. So, we focused on the variability and its effect on the performance using a set of mechanical variables, finger forces in the current study. Nevertheless, we have to admit that the current results of the positive index of the moment stabilization cannot fully be viewed as a purposeful organization by the controller.

Then, what are the benefits of additional constraints such as the moment stabilization into motor tasks? One obvious observation in the current result was that the accuracy and reproducibility of the performance were enhanced when all four fingers were involved, and it seems that the additional moment constraint was activated only with the use of all four fingers. Again, the moment was not dramatically stabilized when two or three fingers (e.g., IM and IMR conditions) were involved. Theoretically, net moment had no effect on the accuracy and precision as the algorithms for the hitting point was programmed in the current study. Theoretical solution space of force-moment task is smaller than space spanned by the Jacobian of one of them. Indeed, the results show that the two variances per dimension, V_UCM_ and V_ORT_, decrease together with the number of fingers, while the synergy indices increase. In the computational point of view, the synergy indices refer to the relative magnitudes of V_UCM_ to V_ORT_ with respect to the total variance (V_TOT_) across multi-attempts. This means that the strength of stability properties (i.e., synergy index) does not depend on the magnitudes of the two variances separately, but on their relative magnitudes^34^. In other words, less flexible combinations of elements (i.e., smaller variances) could be associated with better stability. We know that a unique optimal solution describes a preferred pattern of sharing between the redundant elements. Therefore, less flexible patterns of finger force combinations in the current result may be inclined to the optimal combination of finger forces in case that it minimizes a specific cost function. Thus, it is probable that the finger force combinations are closed to optimal combination without an expense of stability given the condition that more fingers are actively involved in the motor task. Rather, redundant degrees-of-freedom may be a prerequisite to stabilize the given (i.e., net force) and selected (i.e., net moment) performance variables with more converged solutions. Then, what is the nature of cost-function in the current experiment and data set? We do not have the relevant result to answer this question at the moment, and the answer will not be trivial and straightforward. Based on the current results of increased stability index with smaller variance at four finger case, however, we infer that the cost to be minimized may not be energy consumption since it has been mathematically and experimentally proved that the system could not reproduce robust stability during conservation of energy^35^. This speculation is also an important part of a more general movement such as gait since robust stability takes higher priority over energy consumption^35^. In case of sports such as archery, improvement in accuracy and precision of performance is only possible with stabilization of force. From our results, it is also evident that accuracy and precision index is increased with the number of active fingers (i.e., increase in DOFs). Hence, stability takes a higher priority than energy consumption in case of sporting events more than that of a conventional movement used in everyday life. Thus, there may be a trade-off between minimization of energy consumption and stable performance (i.e., extra energy is necessary to resist and maintain stability). The advantage of having an abundant set of elements supports the models of back-coupling feedback loops that describes two separate control processes for defining preferred and flexible sharing patterns^36^. Based on the model and a few current results^13,19^, the optimality and variability may be complimentary ideas in a redundant (abundant) human motor system.

Lastly, in the current study, we reproduced the characteristics of archery performance to examine the mechanism of human multi-elemental control regarding the DOFs of the system. However, the mechanical constraints of the current motor task do not exactly match the real archery task. Previous studies reported that independence of finger actions and sharing patterns depend primarily on the mechanical constraints within the task^37–39^; thus, the effect of different combinations of mechanical constraints on the performance and coordination patterns of finger actions remain to be explored. It has been known that the redundancy emerges at various levels within the human movement system^40–42^. However, the redundancy in the current study was limited to the task redundancy. The extrinsic muscles including flexor digitorum profundus (FDP), superficialis (FDS), and intrinsic palmar muscles, which extend to the phalanges of fingers, are incorporated into the generation of finger flexion force. Thus, a redundant set of muscles to the finger flexion force constructs another layer of motor redundancy. If the non-zero co-contraction of the groups of finger flexors and extensors was observed, this provides another problem of motor redundancy. This is one of limitations of the current study that should be examined in future studies. Nevertheless, it is possible that the electromyography recording from the finger flexors and extensors could reveal the relation of the task redundancy to the muscle redundancy.

## Methods

### Subjects

Eight right-handed young males (age: 29.1 ± 3.9 years, height: 175.3 ± 5.2 cm, mass: 72.8 ± 8.7 kg, hand length: 18.36 ± 0.57 cm, and hand width: 8.37 ± 0.54 cm) were recruited in the study. None of the participants had a previous history of upper extremity injury including forearm, hand, and fingers. Seoul National University Institutional Review Board (IRB) approved the use of customized experimental protocol related to multi-finger pressing tasks and relevant devices (e.g., experimental frame and force transducers). All experimental details were performed in accordance with the relevant guidelines and regulations. The consent was informed, and all participants were requested to sign a consent form approved by the IRB at Seoul National University (IRB No. 1703/002-006).

### Equipment

A set of four six-component force transducers (Nano-17, ATI Industrial Automation, Garner, NC) was used to measure individual finger forces and moments of force for all three axes. The transducers were attached to a vertically oriented aluminum frame. Finely round-shaped caps were inserted into the surface of the transducers (Fig. 6). The position of the panel with the transducers was adjusted along the *x*-axis according to the hand anatomy of individual subjects. Once the position of the panel was adjusted, the panel was mechanically fixed on the table throughout the whole experiment for each participant.

## Experimental Procedure

### Maximal voluntary contraction (MVC) task

The participants were instructed to press on the transducers with all four fingers simultaneously as hard as possible until the maximum total finger pressing force (MVC_TOT_ about the *z*-axis) was achieved. Each trial was performed for 5 s, and the real-time visual feedback of total finger force (F_TOT_) was provided. The maximal individual finger forces (MVC_i_; *i* = {*index*, *middle*, *ring*, *little*}) were captured at the time of MVC_TOT_. The participants performed two consecutive trials, and the average MVC_TOT_ and MVC_i_ values across the two trials were used to set target force levels for the next task.

### Multi-finger force production and release (FPR) task

The participants were asked to produce a steady-state F_TOT_ with multiple fingers followed by a quick release of F_TOT_ in a self-paced manner. The task was a simulation of an archery shooting regarding the sequence of finger force changes (i.e., steady state to release). The experimental conditions of the multi-finger combinations included (1) index-middle (IM), (2) index-middle-ring (IMR), and (3) index-middle-ring-little (IMRL). These combinations of fingers as a set of finger DOFs were assumed to be functionally relevant sets across daily life and sport activity. Ten seconds were given for each trial, which consisted of two phases: (1) steady-state force production and (2) force release phase. For the steady-state phase, it was instructed to maintain steady-state levels of force in both normal (*z*-axis) and tangential (*x*- and *y*-axis) directions within the first 5 s. The magnitude of the reference force (F_REF_) of the *z*-axis was set at 50% of ΣMVC_i_ at corresponding condition (e.g., 50% of {MVC_I_ + MVC_M_} for the IM condition), while the F_REF_ of the *x*- and *y*-axis were set at zero (N). For the force release, the participants were instructed to release finger forces as quickly and smoothly as possible within the next 5 s by translating the forearm and hand toward the trunk while minimizing possible changes in the configurations of individual fingers. The instruction of “*quick*” and “*smooth*” release was for minimizing the release time (i.e., minimum changes in momentum). The initial velocity was computed by equation (1):

1 $${v}_{o}^{j}=\frac{{F}_{t0}^{j}}{\sqrt{k\,m}}-\frac{{\int }_{t0}^{t1}{F}^{j}(t)\,dt}{m},$$

where *j* = {*x*, *y*, *z*}, *k* and *m* represent the coefficient of elasticity of the virtual bow (0.7 N/m) and the mass of the virtual ball as a particle (1 kg), respectively. t_0_ and t_1_ stand for the onset time of release and the time of completion of force release after t_0_, respectively. The horizontal distance from the release point to the virtual target was set at 0.7 (m/N) × ΣMVC_*i*_. Prior to the data collection, the participants performed practice trials for about 30–40 min. For the data collection, the participants performed consecutive 25 trials for each condition. Ten-second break between every two trials and more than 5 min break between the conditions were provided.

### Data analysis

Before variable computation, a digital zero-lag 4^th^-order low-pass Butterworth filter with a cutoff frequency of 10 Hz was applied to the raw force data. Following indices of timing variable were detected within a single trial. t_0_ was defined as the time moment when the first derivative of F_TOT_ (*d*F/*d*t) reached 5% of the first negative peak of *d*F/*d*t. The duration of the steady-state phase was defined as between −1500 ms and −500 ms prior to t_0_. The time of completion of force release (t_1_) was defined as the time moment when F_TOT_ reached 0 N after t_0_.

### Performance indices

#### Root mean squared error (RMSE)

Root mean squared error (RMSE) was computed using equation (2) during the steady-state phase.

2 $$RMS{E}_{NORM}^{j}=(\sqrt{{\sum }^{n}{({F}^{j}-{F}_{REF}^{j})}^{2}/n})/{F}_{REF}^{j},$$

where, *j* = {*x*, *y*, *z*}, and *n* represents the number of data samples during steady-state force production. F and F_REF_ represent the produced total forces by the participants and the total reference force, respectively.

#### *Release time* (*RT*)

RT was defined as time duration between t_0_ and t_1_. Further, we quantified average (RT_AVG_) and standard deviation (RT_SD_) of RTs across repetitive trials in each participant and condition.

#### *Accuracy* (*ACI*) *and precision index* (*PRI*)

The accuracy index (ACI) was quantified as an average Euclidean distance between the hitting position of the virtual ball and the center of the virtual target across repetitive trials using equation (3). The precision index (PRI) was quantified as an average Euclidian distance between the hitting position of the virtual ball and mean hitting position across repetitive trials using equation (4). The ACI and PRI were further normalized by the horizontal distance from the release point to the virtual target.

3 $$ACI=\frac{{\sum }_{i=1}^{n}\sqrt{{({x}_{{i}}-{x}_{CENT})}^{2}+{({y}_{i}-{y}_{{CENT}})}^{2}}}{n}$$

4 $$PRI=\frac{\sum _{i=1}^{n}\sqrt{{({x}_{i}-{x}_{MEAN})}^{2}+{({y}_{i}-{y}_{MEAN})}^{2}}}{n},$$

where, *i* = each trial, {*x*_*i*_, *y*_*i*_}: *x*- and *y*-coordinate of the hitting position for each trial, {*x*_*CENT*_, *y*_*CENT*_} = *x*- and *y*- coordinate of the center of the virtual target (0 mm), {*x*_*MEAN*_, *y*_*MEAN*_} = *x*- and *y*-coordinate of the mean hitting position for all trials.

### Multi-finger synergy indices

The framework of uncontrolled manifold (UCM) approach was conducted to quantify the synergy indices of stabilizing two sets of performance variables, resultant force (F_TOT_) and resultant moment of force (M_TOT_), separately. The repetitive trial data were aligned with respect to t_0_. The elemental variables in the current analysis were finger forces, and the performance variables included F_TOT_ and M_TOT_. For M_TOT_ computation (i.e., the product of finger forces and their lever arms), we assumed that the center of rotation was located at the mid-point between two lateral fingers on the level of the surface of the sensors along the *y*-axis, and the lever of individual finger forces were fixed. Note that M_TOT_ was not measured but computed from linear combinations of finger forces multiplied by the fixed lever arm vector. There is no actual pivot of rotation; thus, the participants were free to vary fingertip displacement, which had no mechanical effect on the performance indices. The manifold was found by computing the null space of the Jacobian of the transformation (i.e., linearly approximated null space spanned by the basis vector). The time series of variances within two subspaces, V_UCM_ and V_ORT_ (equation 5) across repetitive trials were computed for each performance variables and each condition. The UCM was defined as an orthogonal set of the unit vectors in the space of finger forces that did not change the given performance variables. The other subspace (ORT) was the orthogonal vector to the UCM. The time function of synergy index, ΔV(*t*), was further computed using equation (5) (see Latash *et al*.^31^ for computational details). Note that the variances in equation (5) were further normalized by the DOFs of the corresponding subspace. This process was necessary to make comparable indices across subspaces of different dimensions (i.e., DOFs).

5 $${\rm{\Delta }}V(t)=\frac{{V}_{UCM}(t)/DO{F}_{UCM}-{V}_{ORT}(t)/DO{F}_{ORT}}{{V}_{TOT}(t)/DO{F}_{TOT}}$$

Lastly, the ΔVs were log-transformed using the Fischer transformation applied for the computational boundaries (i.e., −2 to +2 for the IM, from −3 to +1.5 for the IMR, from −4 to +1.33 for the IMRL condition).

### Statistical analysis

The data are presented as means and standard error. Repeated measured ANOVAs with factors *Finger* (four levels: index, middle, ring and little), *Axis* (three levels: *x*-, *y*-, and *z*-axis), *Finger DOF* (three levels: IM, IMR, IMRL), and *Variance* (two levels: UCM and ORT) were used. Notably, we explored how the main outcome variables (RMSE_NORM_, RT_AVG_, RT_SD_, ACI, PRI, ΔV_F_, ΔV_M_) were affected by the factors. The factors were selected based on particular statistical tests. For the repetitive measures of the force values of three axes (F_X_, F_Y_, F_Z_), the intra-class correlation coefficents (ICC) as an index of test-retest reliability were estimated for IM condition (ICC = 0.954 for F_X_, *p* < 0.001; ICC = 0.977 for F_Y_, *p* < 0.001; ICC = 0.997 for F_Z_, *p* < 0.001), IMR condition (ICC = 0.927 for F_X_, *p* < 0.001; ICC = 0.986 for F_Y_, *p* < 0.001; ICC = 0.999 for F_Z_, *p* < 0.001), and IMRL condition (ICC = 0.981 for F_X_, *p* < 0.001; ICC = 0.969 for F_Y_, *p* < 0.001; ICC = 0.996 for F_Z_, *p* < 0.001). Mauchly’s sphericity test was used to confirm the assumptions of sphericity, and the Greenhouse-Geisser correction was used when the sphericity assumption was rejected. For the post-hoc test, multiple pairwise comparisons with Bonferroni correction was conducted. Linear regression analysis was used to examine the relations between the synergy indices (ΔV_F_) and performance indices (ACI and PRI). All statistical significance level was set at *p* < 0.05.

## Data Availability

The datasets measured during the current experiments are available from the corresponding author (Jaebum Park) on reasonable request.
